# ∆Np73 is capable of inducing apoptosis by co-ordinately activating several BH3-only proteins

**DOI:** 10.1042/BSR20150039

**Published:** 2015-06-11

**Authors:** Dámaso Sánchez-Carrera, Mikel García-Puga, Lucrecia Yáñez, Íñigo Romón, Carlos Pipaón

**Affiliations:** * Laboratorio de Hematología Molecular, Servicio de Hematología y Hemoterapia, Hospital Universitario Marqués de Valdecilla–IDIVAL, Santander, Spain

**Keywords:** ∆Np73, apoptosis, BH3-only proteins, Fanconi anaemia, p53, specificity protein 1 (Sp1)

## Abstract

Inactivation of p53 is one of the most relevant events in human cancer, since it allows transformed cells to escape their own proliferation control and leave them irresponsive to drugs that aim to damage their DNA. When p53 falls, other members of its family may become targets to attack tumoural cells. p73 has shown capacity to mediate these attacks. However, its N-terminal truncated isoforms have been associated with oncogenesis due to their capacity to act as dominant negatives of p53 and the transactivation (TA) isoforms of p73. We previously found a relationship between the overexpression of N-terminus-truncated p73 isoform (∆Np73) and that of the proapoptotic gene Bcl-2-interacting killer (BIK). In the present report we demonstrate that ∆Np73-α has the capacity to induce apoptosis through the co-ordinated activation of a group of genes harbouring GC-rich elements in their regulatory regions. ∆Np73-α synergizes with specificity protein (Sp1) on these elements but the overall response of these genes probably depends on the additional presence of consensus p53 elements. We explore the domains of ∆Np73-α involved in this transactivation capacity and found divergences with the previously described functions for them. Moreover, we found that the transforming mutation V12 of HRas impairs this transactivation capacity of ∆Np73-α, further supporting the anti-tumoural function of this later. Our data add complexity to the action of p73 on the induction of apoptosis and tumourogenesis, opening new interpretations to the expression profile of p73 isoforms in different human neoplasias.

## Abstract (precis)

∆N isoforms of p73 interfere with p53 action, acting as oncogenes. In the present study, we report that ∆Np73 is induced after DNA damage and is also capable of co-ordinately activating pro-apoptotic genes thus mediating p53 action after DNA damage.

## INTRODUCTION

The p53 family of transcription factors are long known for its function in the preservation of the genomic information, activating genes involved in the repair of the DNA, but also stopping proliferation and inducing a controlled cell death when the damage exceeds a certain threshold. These factors rapidly accumulate in response to physical or chemical DNA insults, including radio- and chemo-therapies. Mutations in p53 have been described for virtually all human tumours, making them scape their own control systems and making inefficient all the therapeutic efforts aimed to promote these later. This is the main attractive of other members of the p53 family that could mediate a chemotherapy response in the absence of a functional p53.

p73 is not commonly mutated in human cancer, but is known to be needed for an optimal apoptotic response in co-operation with p53 and p63 [1]. Alternative promoter usage and splicing produces a plethora of p73 isoforms. Alternative usage of the exons coding for the C-terminus of the protein produce at least seven variants termed with Greek letters (α–η). Those exons code for a sterile α motif (SAM) domain and a repressive domain located at that end of the protein [2]. On the other hand, the TP73 gene contains an alternative promoter located in the third intron that gives rise to N-truncated (∆N) isoforms [3]. Combinations of N- and C-end variations are possible, so the number of isoforms is quite large. A transactivation (TA) domain was mapped at the amino end of the protein, so those isoforms carrying the complete N-terminus are noted as p73 containing the TA domain (TAp73). Since p53 family members bind to DNA as tetramers, ∆N isoforms, lacking the TA domain but harbouring a DNA-binding domain and an oligomerization domain (OD), act as dominant negatives of the TA isoforms, binding to them and competing for the binding sites, but impairing transactivation. Thus, by interfering the proapoptotic and cell cycle regulatory functions of p53 and TAp73, N-terminus-truncated p73 isoform (∆Np73) acts as an oncogene and it has been reported to be overexpressed in several tumours [4]. However, many times those same tumours also express high amounts of TAp73, somehow suggesting that ∆Np73 has other functions than just interfering with the action of TA isoforms.

Supporting this idea, it has been reported that overexpression of ∆Np73-α in a p53 wild-type background does not affect cell proliferation or the response to DNA damage. Moreover, when injected into nude mouse, ∆Np73-α-overexpressing cells does not promote a more aggressive phenotype of the tumours generated [5]. ∆Np73 has actually been shown to be able to induce gene transcription on its own [6–10], although the mechanisms are still not fully understood. Liu et al. [7] described that the 13 unique N-terminal residues of ∆Np73-β, together with the adjacent PXXP motifs, constitute a novel TA domain, mediating the capacity of the isoform to suppress cell growth and promote apoptosis. Interestingly, ∆Np73-α was found to be inactive in these experiments. The SAM domain and C-terminus of the TAp73-α isoform have been reported to exert an inhibitory effect over its TA domain, suppressing its interaction with p300/CBP co-activators [2]. Toh et al. [9] reported a new mechanism by which ∆Np73-β, but not α, could activate the transcription of the pro-survival caspase-2S through a GC-rich specificity protein (Sp1)-binding site in its promoter, a mechanism also used by the TAp73 isoforms [9,11].

An exacerbated activation of p53 has been reported to be at the basis of the progressive bone marrow failure in Fanconi anaemia (FA) patients, and the sensitivity of their hematopoietic cells to genotoxic agents [12]. We found that the increased p53 stabilization in cell lines derived from FA patients is paralleled by a ∆Np73 transcriptional activation [10], so we wanted to explore the functional relationship between these two proteins in the induction of apoptosis in these cells. The results would also apply to other pathologies with an elevated expression of both p53 or TAp73 and ∆Np73, like chronic lymphocytic leukaemia or ovarian cancer [13,14]. Our results demonstrate that ∆Np73 can actually mediate the p53 induction of apoptosis through its binding to GC-rich elements in several pro-apoptotic genes.

## EXPERIMENTAL

### Cell culture and cytometry

Fibroblasts derived from FA-A patients and 293FT human embryonal kidney cells, both transformed with the SV40 large T antigen, were maintained in Dulbecco's modified Eagle's medium (DMEM) (BioWest) supplemented with 10% heat-inactivated foetal calf serum and grown in a humidified 5% CO_2_-containing atmosphere at 37ºC. pCR3.1-∆Np73-α, pECFP Cyan and shRNA vectors (Sigma) were transiently transfected into the cells using Superfect (Qiagen) according to the manufacturer's directions. Cells entering apoptosis were immunostained with an anti-Annexin V-FITC antibody (Immunostep) and propidium iodide, and then analysed by flow cytometry.

### RT-PCR analysis

Total RNA was prepared using TRIZOL reagent (Invitrogen). To assess mRNA expression, a semiquantitative reverse transcriptase–polymerase chain reaction (RT-PCR) method was used. For the RT reaction, RNA (5 μg) was primed with random hexamers and reverse transcribed with Superscript MMLV reverse transcriptase (Invitrogen) in a 20 μl volume following the manufacturer's instructions. The generated cDNA was amplified using primers for human ∆Np73 (5′-CGCCTACCA-TGCTGTACGTC and 5′-GTGCTGGACTGCTGGAAAGT), TAp73 (5′-TCTGGAACCAGACAGCACCT and 5′-GTGC-TGGACTGCTGGAAAGT), Bcl-2-interacting killer (BIK) (5′-GACCATGGAGGTTCTTGGCA and 5′-AGGCTCACGTC-CATCTCGTC), p53 up-regulated modulator of apoptosis (PUMA) (5′-ACGACCTCAACGCACAGTACG and 5′-TGG-GTAAGGGCAGGAGTCC), phorbol-12-myristate-13-acetate-induced protein 1 (NOXA) (5′-AGCTGCGTTTCACCAGGG and 5′-TCCAGCTACTTGCACTTGTTCCT), early growth response 1 (EGR1) (5′-TGACCGCAGAGTCTTTTCCT and 5′-TGGGTTGGTCATGCTCACTA), p21 (5′-GAGGCACTCAGA-GGAGGCGCCAT and 5′-CACACGCTCCCAGGCG-AAGTC) and β2- microglobulin (B2M) (5′-GAGACA-TGTAAGCAGCATCA and 5′-AGCAACCTGCTCAGA-TACAT). After 30 amplification cycles, except for B2M (25), the expected PCR products were size-fractionated on to a 2% agarose gel and stained with ethidium bromide.

### Western blot analysis

To prepare whole cell lysates, cells were collected by centrifugation and washed once with PBS and then lysed in EBC lysis buffer (Cold Spring Harb Protoc; 2006; doi:10.1101/pdb.rec8856) [50 mM Tris (pH 8.0), 120 mM NaCl, 0.5% Nonidet P-40] supplemented with aprotinin (11.5 μg/ml), leupeptin (11.5 μg/ml) phenylmethylsulfonyl fluoride (50 μg/ml), NaF (100 mM) and Na ortovanadate (0.2 mM). Protein concentration was determined by BCA following the manufacturer's instructions (G Biosciences). Proteins (25 μg) were resolved in SDS/PAGE and transferred to PVDF filters. Blots were incubated with a mouse antibody against p73 (ER-15) (Thermo-Pierce) and then incubated with an anti-mouse antibody conjugated with horseradish peroxidase (Santa Cruz). Bound antibody was detected by a chemiluminescence assay (GE Healthcare).

### Gene reporter assay

A genomic PCR fragment of 1038 bp (−998 to +40, referred to as the initiation of transcription) from the 5′ regulatory region of BIK was amplified with specific primers and cloned into the pGL2basic luciferase reporter vector (Promega). Deletions of this original construct were obtained by amplification using specific primers and cloning into pGL2basic. Similarly, the −666 to +22 sequence around the transcription start site of EGR1, and the −1235 to +142 sequence around the transcription start site of NOXA were amplified from human genomic DNA and cloned into the same reporter vector. p21 −2326 to +11 luciferase construct was described somewhere else [15]. The short responsive elements and their mutants were synthesized (Thermo Fisher Scientific), annealed and cloned upstream of a TATA box in pGL2basic. Human ∆Np73-α and −β ORFs were amplified using specific oligonucleotides and cloned into pCR3.1 (Invitrogen). These constructs were the template to generate the indicated truncations of the proteins, using specific primers. 293FT cells were cotransfected with 2 μg of the reporter constructs, 1 μg of the adequate expression vectors and 0.2 μg of pEF-BOS-β-galactosidase in triplicate using the calcium phosphate technique. Luciferase and β-galactosidase activities were assessed 24–48 h following transfection using reagents from Promega and Thermo Fisher Technologies, respectively. The luciferase activities were normalized to β-galactosidase to obtain relative luciferase activity.

## RESULTS

### Cross-linking agents induce ∆Np73

We have previously observed a concomitant activation of the N-terminal truncated isoform of p73 and the pro-apoptotic gene BIK in FANCC (FA complementation group C gene)-deficient lymphoblastoid cell lines [10]. We wanted to test whether ∆Np73 exacerbated induction is a common feature to other FANC-deficient cell lines. We treated immortalized fibroblasts derived from FA group A or group D2 patients with cisplatin or mitomycin C (MMC). FA cells are highly sensitive to the DNA damage caused by these drugs. At the concentrations used, both drugs induced an accumulation of the messenger of ∆Np73 but not that of the corresponding TA isoform (Figure 1). When the FANC-deficient cells were corrected by transduction of the corresponding wild-type FANC gene cDNA, ∆Np73 mRNA induction was dampened, pointing to DNA damage as its cause. Interestingly, the messengers coding for several proapoptotic proteins like BIK, NOXA and PUMA paralleled the expression induction of ∆Np73, suggesting a possible regulatory bond between them.

**Figure 1:**
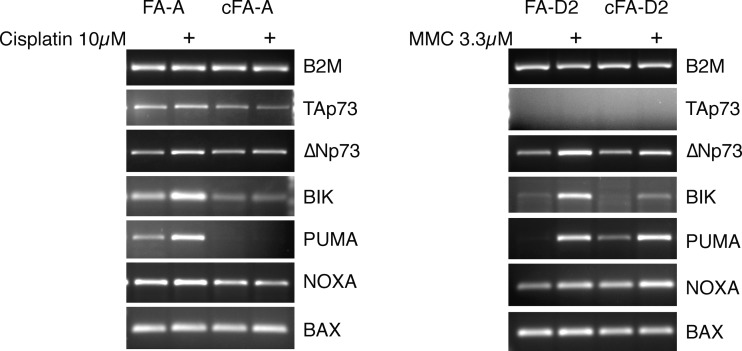
∆Np73 is induced by DNA damage Immortalized fibroblasts in culture were grown in the presence of 10 μM cisplatin or 3.3 μM MMC for 48 h as indicated and their RNA was analysed by semiquantitative RT-PCR. Cisplatin and MMC induce ∆Np73-α mRNA and a group of apoptosis and cell cycle-controlling genes in cells defective in the FA-BRCA DNA-repair pathway (FA-A or FA-D2). This induction is impaired when the cells were reverted with the corrective gene (cFA-A or cFA-D2).

### BIK promoter is activated by ∆Np73 through a GC-rich element bound by Sp1

In order to further investigate the regulation of BIK transcription by ∆Np73 we studied the promoter region of the gene by luciferase assays. We previously cloned up to 1 kb upstream of the BIK transcriptional start site controlling the luciferase gene expression and observed that p53 overexpression had no effect on BIK promoter activation. In contrast, ∆Np73-β and specially ∆Np73-α, produced a robust activation of BIK promoter [10]. We then seek to identify the element within BIK promoter mediating ∆Np73 activation. To that end we constructed reporter plasmids with serial deletions of the promoter. When the region between positions −96 and −38 was deleted, a strong reduction both in basal and ∆Np73-induced activation was observed (Figure 2A). The analysis of the sequence between positions −96 and the transcriptional start site showed no consensus response element for p53 family members. However, a cluster of three Sp1 response elements reside between positions −72 and −38 (Figure 2B), somehow explaining the reduction in basal promoter activity when this region is deleted. p53 and Sp1 families of transcription factors have been reported to physically and functionally interact in the induction of cell-cycle arrest and proapoptotic genes [16]. Thus, to investigate whether the region between −72/−38 is mediating the activation of BIK promoter by ∆Np73, we cloned this region upstream of a TATA box, controlling the transcription of the luciferase gene, and cotransfected it along with an expression vector for ∆Np73-α into 293FT cells. ∆Np73-α and to a lesser extent, ∆Np73-β induced a striking activation of the luciferase gene whereas neither p53 nor the TA forms of p73 had any effect (Figure 2C).

**Figure 2:**
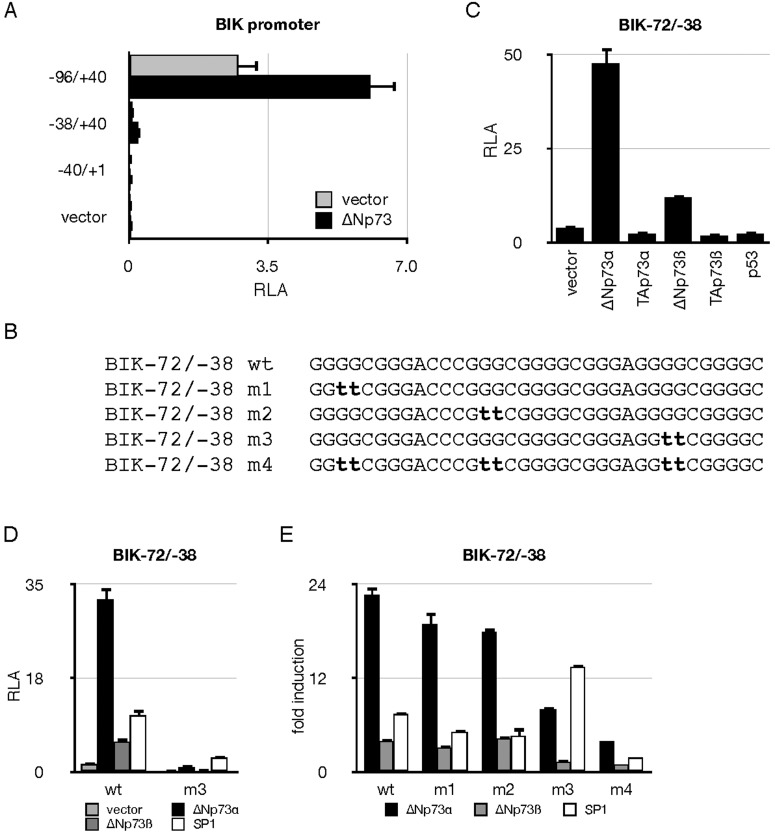
Induction of BIK promoter by ∆Np73-α **(A**) The region between positions −96 and −40 mediates the activation of BIK promoter by ∆Np73-α. A luciferase reporter controlled by the indicated portions of the BIK promoter was transfected along with an expression vector for ∆Np73-α (black bars). The luciferase activity relative to the activity of an internal β-galactosidase control [relative luciferase/galactosidase activity (RLA)] in the cell extracts is depicted. (**B**) Sequence of the region between positions −72 to −38 relative to the transcriptional start of the human BIK gene, and that of the different mutants generated. (**C**) ∆N isoforms of p73 but neither TAp73 nor p53 activate the region between positions −72 and −38 of BIK promoter. This region confers responsiveness to a heterologous basic promoter driving the luciferase gene. RLA is represented. (**D**) RLA of the wild-type and mutant 3 (m3) BIK-72/-38 constructs in response to ∆Np73-α (black bars), ∆Np73-β (dark grey bars) or Sp1 (white bars). Mutation 3 greatly impairs basal activity as well as its induction by ∆Np73-α and −β. (**E**) Induction of luciferase activity in the wild-type and mutant BIK-72/-38 constructs by ∆Np73-α (black bars), ∆Np73-β (dark grey bars) or Sp1 (white bars). Although mutations 1 and 2 barely affect the induction of this region by ∆Np73-α and −β, mutation 3 clearly reduces it. Contrarily, m3 potentiates the activation of this region by Sp1. Fold induction was calculated in relation to empty vector-transfected control cells.

### The GC-rich element in BIK promoter harbours responsive elements for Sp1 and ∆Np73

The analysis of the region between −72 and −38 of the BIK gene promoter revealed at least three different putative Sp1-binding sites. Sp1 can certainly activate transcription through this region, as our cotransfection experiments showed (Figures 2D and 2E). Moreover, Sp1 and ∆Np73 synergistically transactivate this element of BIK promoter (see Figure 3D). We aimed to mutate each of the putative binding sites individually or together to study its effect on ∆Np73-α transactivation. Mutants m1, m2 and m3 change a critic CC dinucleotide to TT within three of the putative Sp1-binding sites (Figure 2B). Mutant m4 meets all three changes together. All four mutants showed reduced basal activity when compared with the wild-type construct. However, we found differences in their capacity to respond to Sp1 or ∆Np73-α. Although m1 and m2 retained most of the activation capacity by ∆Np73, m3 profoundly impaired it (Figure 2E). On the other hand, none of the single site mutations greatly affected transactivation by Sp1, being this only affected when all three were mutated together (m4). Moreover, m3 showed a greater activation in response to Sp1 than m1 or m2, suggesting some kind of compensatory activation through the other sites (Figure 2E). In agreement with this, we could observe that the 18 bp sequence between positions −56 and −38 retain the capacity to respond both to ∆Np73 and Sp1 (see Figure 3B).

**Figure 3:**
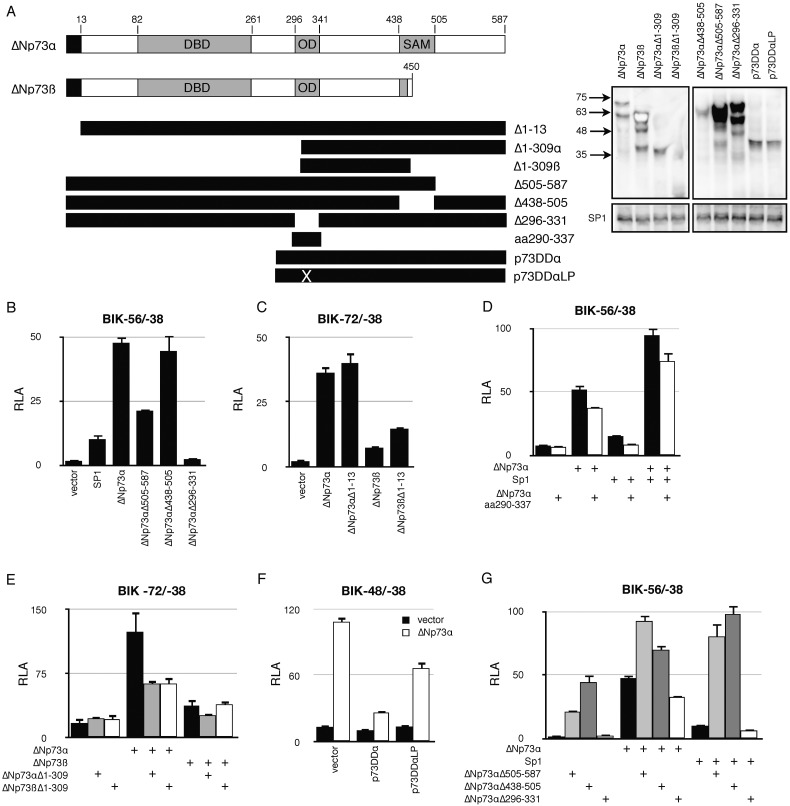
Analysis of ∆Np73 domains important for its activation of BIK (**A**) Scheme showing the main domains of ∆Np73-α and −β, as well as the truncations used in our experiments. At the right hand a western blot shows the expression of the different constructs. The same blot was stripped and re-incubated with an anti-Sp1 antibody. (**B**, **C**). Activation of the responsive element within BIK promoter by ∆Np73 lacking different important domains of the protein. Truncation of the C-terminus or the ODs greatly affects the transactivation capacity of ∆Np73-α (B). Elimination of the SAM domain or the 13 N-terminal isoform-distinctive residues has virtually no effect (B, C). RLA is represented. (**D**) Dominant negative effect of the OD of p73 over ∆Np73-α and Sp1 in the activation of the responsive element of BIK. An expression vector for the region between amino acids 290 and 337 of ∆Np73-α was transfected along with Sp1 or the full length ∆Np73-α to analyse their effect on the activation of the response element of BIK. RLA in the cell extracts is depicted. (**E**) Deletion of the N-terminal and DNA-binding domains of ∆Np73-α and −β abolish their transactivation capacity, but those truncated forms act as dominant negatives of the full length proteins. The indicated constructs were cotransfected and the RLA in the cell extracts determined. (**F**) p73DDα, embracing amino acids 327–636 of p73α [26], act as dominant negative of ∆Np73α, but the mutation L371P within its OD (p73DDαLP) impairs this effect. (**G**) ∆Np73-α proteins lacking the SAM or the C-terminal domains are still capable of synergizing with Sp1 in the activation of the responsive element in the BIK promoter. Contrarily, deletion of the OD of the protein completely abolish its transactivation and Sp1-synergizing capacities. RLA in the cells transfected with the indicated constructs is represented.

### Transactivation by ∆Np73 requires its DNA-binding and oligomerization domains

We then seek to identify the domains within the ∆Np73-α structure needed for its activation of BIK. To that end, we performed truncations of functionally relevant domains of ∆Np73 to analyse their effect on its TA capacity. A scheme of such truncations is detailed in Figure 3A. As expected by the differential activation potential of the α- and β-isoforms, truncation of the extreme C-terminus of ∆Np73-α greatly impaired its TA potential of BIK (Figure 3B). The SAM domain, located between residues 438 and 505, seemed to be dispensable for this activation, as well as the most N-terminal 13 amino acids, distinctive of the ∆N forms (Figures 3B and 3C). Contrarily, deletion of the OD, located between residues 296 and 341, completely abrogated the TA capacity of ∆Np73 (Figure 3B). Accordingly, constructs overexpressing this domain acted as dominant negatives over BIK activation (Figures 3D and 3E), and the mutation L371P impairs such dominant negative effect (Figure 3F). Interestingly, ∆Np73-α lacking either the SAM or the C-terminus still synergizes with Sp1 in the activation of BIK promoter, suggesting these domains do not participate in the interaction with Sp1 (Figure 3G).

### Other apoptosis-related genes are up-regulated by ∆Np73 through similar Sp1 sites

Once we demonstrated that ∆Np73 is able to activate BIK transcription through Sp1 responsive elements, we investigated whether other cell cycle-arresting or apoptosis-promoting genes may also be co-ordinately regulated by this isoform. We focused on genes harbouring Sp1 responsive elements similar to that of BIK. We tested the regions embracing positions −1235 to +26 of NOXA, −666 to +22 of EGR1 and −2326 to +11 of p21. In cotransfection experiments using luciferase constructs, we observed that ∆Np73-α, but not TAp73-α, induced a strong activation of EGR1 promoter. On the contrary, p21 and NOXA promoters were activated by TAp73 and repressed by ∆Np73, as previously reported (Figure 4A). We also analysed whether isolated GC-rich elements from these promoters could be activated by ∆Np73. Several elements located upstream of the exon 1a of PUMA were also analysed, since they have been reported to mediate its activation by p73 [17]. The regions shown in Figure 4B were cloned upstream of a TATA box and cotransfected along with several expression vectors. We could demonstrate that these 11 bp are strongly activated by ∆Np73-α in BIK and PUMA, and to a lesser extent in NOXA (Figure 4C). It is noteworthy that BIK, PUMA and NOXA contain several of these GC-rich elements in tandem, suggesting a possible amplification effect. Indeed, a wider region embracing the sequence between positions −60 and −20 of NOXA mediates a strong activation by ∆Np73-α, but not by TAp73-α or p53 (Figure 4D). Altogether, these results support the idea that ∆Np73-α is able to co-ordinately activate genes involved in cell cycle arrest and apoptosis, although in a way strongly dependent of the promoter and probably the cellular context.

**Figure 4:**
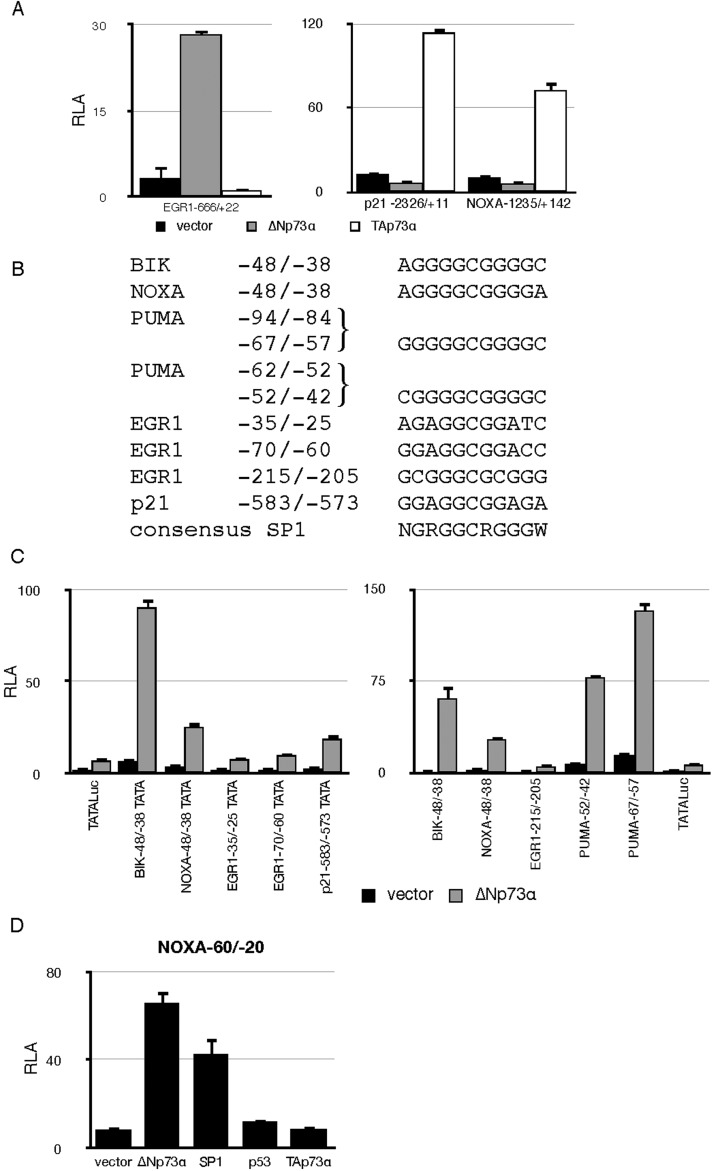
∆Np73 induces other cell cycle-arresting and apoptosis-promoting genes (**A**) Effect of ∆Np73-α on the regulatory regions of NOXA, EGR1 and p21 genes. Luciferase constructs containing the indicated regions of the genes were tested for their responsiveness to ∆N (grey bars) or TA (white bars) isoforms of p73-α. Like BIK, EGR1 promoter was greatly activated by ∆Np73-α and repressed by the TA isoform. p21 and NOXA regulatory regions showed the opposite behaviour. (**B**) Sequences of the candidate regions to respond to ∆Np73-α found in different genes. The numbers refer to the positions relative to the transcription start site, with the exception of PUMA, that are relative to the beginning of exon 1a. (**C**) RLA in cells transfected with an expression vector for ∆Np73-α (grey bars) or an empty vector (black bars) along with a luciferase constructs driven by the candidate sequences. (**D**) The GC-rich region between positions −60 to −20 of NOXA promoter, containing several Sp1 consensus elements, mediates a specific response to ∆Np73-α, but is neither activated by p53 nor by the TA isoform of p73-α.

### Enforced expression of ∆Np73-α induces apoptosis

Our data demonstrate that ∆Np73-α is capable of activating GC-rich elements in the regulatory regions of certain apoptosis-promoting genes. Our luciferase experiments suggest that the effect of these elements on the overall expression of those genes might depend on their promoter context, so we aimed to study which of the genes are indeed induced when ∆Np73-α is overexpressed in a cell line. Transient transfection of ∆Np73-α into 293FT cells induced an accumulation of PUMA, NOXA, EGR1 and BIK mRNAs (Figure 5A). The activation of these proapoptotic genes should lean the survival balance of these cells toward apoptosis. Indeed, an increase in Annexin V immunostaining was detected 24 h after the transfection of ∆Np73-α (Figure 5B). Moreover, when shRNA interference constructs against BIK, NOXA or PUMA were cotransfected along with ∆Np73-α, the increase in Annexin V positive cells was impaired, indicating that those genes mediate ∆Np73-α action on apoptosis (Figure 5C).

**Figure 5:**
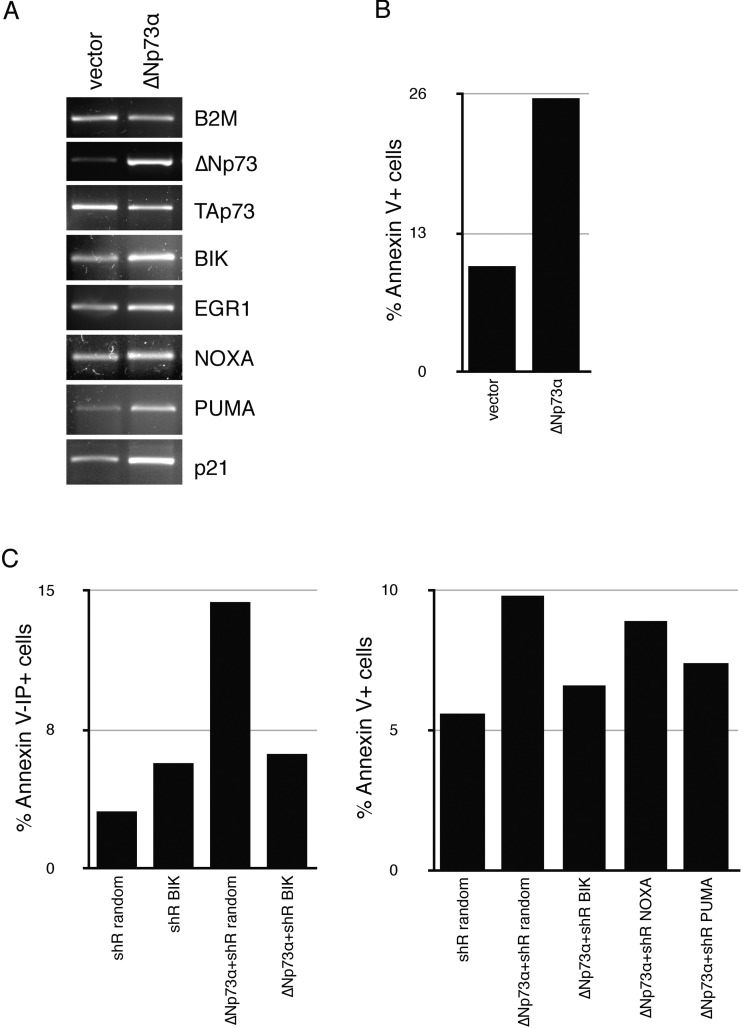
Enforced expression of ∆Np73-α induces apoptosis (**A**) Transient transfection of an expression vector for ∆Np73-α into 293FT cells induces the mRNA expression of several proapoptotic genes. (**B**) Overexpression of ∆Np73-α induces an increment in cells entering apoptosis. 293FT cells transiently transfected as in **A** were analysed for the induction of apoptosis by anti-Annexin V and propidium iodide immunostaining. A representative experiment is shown. (**C**) Interference with BIK expression abolishes the apoptosis induced by ∆Np73-α. Cotransfection of shRNA constructs against the indicated proapoptotic genes along with ∆Np73-α reduces the number of cells immunostained for Annexin V.

### Oncogenic mutants of HRas impair BIK activation by ∆Np73-α

Several papers have reported the key role of oncogenic Ras mutants in the abrogation of the p73 tumour suppressor functions during transformation of primary cells [18]. Since we observed an anti-oncogenic behaviour of the ∆Np73 isoforms, we wanted to address the effect of HRasV12 in this context. Cotransfection of HRasV12 with the reporter construct containing the ∆Np73 responsive element induced an increase in the luciferase activity of the cells (Figure 6A). This effect is probably mediated by Sp1, since a shorter responsive region, lacking some of the Sp1-binding sites, had a weaker response to mutated Ras (Figure 6B). Conversely, HRasV12 impaired the activation by ∆Np73-α of the element in BIK promoter (Figures 6A and 6B). Noteworthily, when the C-terminal region of ∆Np73-α is removed, the inhibitory effect of RasV12 is reduced, underlining the key role of this domain in the TA activity of the protein (Figure 6B). We observed a similar response when using the region between positions −60 and −20 of NOXA promoter driving the reporter (data not shown). We then investigated how HRasV12 affects the induction of apoptosis mediated by ∆Np73-α. As shown in Figure 6C, cotransfection of HRasV12 reverted the apoptosis induced by ∆Np73-α, as measured by Annexin V-IP immunostaining.

**Figure 6:**
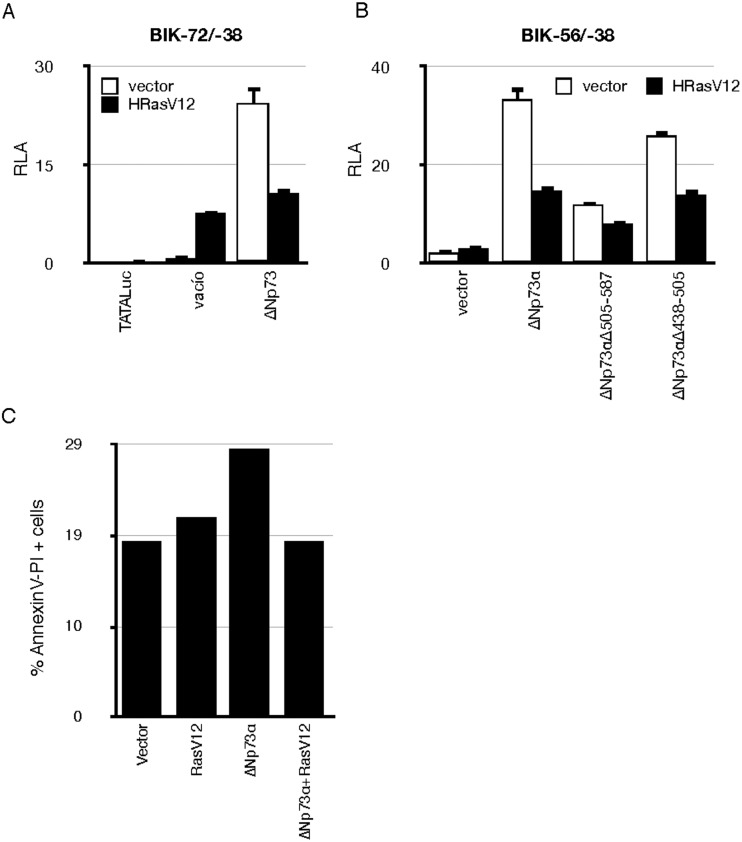
Oncogenic HRasV12 interferes the proapoptotic capacity of ∆Np73-α (**A**) HRasV12 (black bars) activates the basal activity of the ∆Np73 responsive element in BIK promoter, but impairs its induction by ∆Np73-α. Expression vectors for HRasV12 and ∆Np73-α were cotransfected along with the reporter construct containing the region from −72 to −38 of BIK promoter, and RLA was measured. (**B**) Deletion of the C-terminal region of ∆Np73 (∆505–587) impairs the inhibitory effect of HRasV12 over it. Expression vectors for the indicated constructs were cotransfected with a −56 to −38 BIK reporter construct. RLA is depicted. (**C**) Cotransfection of HRasV12 reverted the apoptosis induced by ∆Np73-α. When HRasV12 is cotransfected with ∆Np73-α, the percentage of Annexin V-IP positive cells is reduced to that in cells transfected with empty vectors.

## DISCUSSION

Although p73 is not commonly mutated in human cancer, its function as tumour suppressor has been largely documented. Like p53, p73 is a transcription factor and its TA domain has been mapped to the N-terminal domain of the protein. Indeed, alternatively transcribed or spliced p73 isoforms lacking this N-terminus act as dominant negatives of the TA forms, since these factors need to oligomerize to activate transcription [19]. However, previous reports demonstrated that ∆Np73-β still retains the capacity to activate certain genes [6] and induce cell cycle arrest through a different TA domain comprising its distinctive N-terminal 13 amino acids together with the PXXP motifs [7]. We observed that ∆Np73 mRNA is strongly induced in FANC-deficient cells after DNA damage and we wondered what its function would be in this context. Bone marrow failure in FA patients has been attributed to an aberrant p53 stabilization in hematopoietic stem cells [12] and indeed p53 activates the second promoter of the p73 gene, that generates ∆Np73 [10]. The proapoptotic gene BIK was also induced in FA cells after DNA damage but p53 was not able to activate its promoter. Surprisingly, our data suggest that ∆Np73 is not participating in a negative feedback loop on this promoter but rather mediating p53 actions in inducing apoptosis.

In the present work, we seek to identify the responsive element within the BIK gene regulatory region mediating its activation by ∆Np73-α. Of note, no consensus p53-family responsive elements were obvious by sequence analysis, so we carried out a progressive deletion functional analysis of the promoter. These analysis identified a region between positions −72 to −38 which deletion impairs both basal and ∆Np73-α-induced activities. This region embraces several Sp1 responsive elements as we could demonstrate by cotransfection of an expression vector for this factor. We could also demonstrate that this region is capable of conferring ∆Np73 responsiveness to an heterologous basic promoter, but is unresponsive to the p73 TA isoforms or p53. To the best of our knowledge, this is the first report demonstrating the distinct transactivation capacity of ∆Np73-α through Sp1 responsive elements. That GC-rich region of the BIK promoter harbours at least three putative Sp1 responsive elements with slight variations in their sequence. Our mutational analysis demonstrates that the most proximal of them is critical for the activation of BIK by ∆Np73. The analysis of homologous regions in other cell cycle-controlling or apoptosis genes further confirms the strict sequence requirements for this effect to occur. Besides, the sequence slightly diverges from the consensus Sp1-binding site, as the adenosine at position −38 of the NOXA promoter marks a clear difference in the response, and a C at that position seems to be needed for maximal activation.

On the other hand, our data reveal a capacity of ∆Np73-α to induce apoptosis never reported before. ∆N isoforms of the TP73 gene have hitherto been related to the inhibition of the tumour suppressing activity of p53 and the TAp73 isoforms, either by direct interaction with them, acting as dominant negatives [20], or by activating pro-survival proteins like caspase-2S [9], thus being considered as oncogenes. The finding of ∆Np73 isoforms being overexpressed in several tumours supported this vision [13,21]. However, in several cases these tumours also show high levels of TAp73 isoforms, as in ovarian cancer and chronic lymphocytic leukaemia [13,14]. These observations suggest that ∆N isoforms may have other functions than the simple antagonism on the TA isoforms. Indeed, several phenotypes observed in p73 −/− mice are not reproduced by either of the TA or ∆N specific mutant models, suggesting that both isoforms compensate for each other in certain functions [22–24]. In this respect, several groups, including ours, reported transactivation capacities for the ∆Np73 isoforms [6–10]. In the present study, we provide a mechanism by which ∆Np73-α is able to induce apoptosis, activating the transcription of BIK. Moreover, ∆Np73-α co-ordinately transactivates the promoter of several other apoptosis-promoting genes through Sp1 responsible elements. Since overexpression of ∆Np73 is not eliciting the accumulation of Sp1 (see Figure 3), a mechanism involving ∆Np73α-Sp1 interactions seems plausible. It is long known the interaction between members of these two families of transcription factors, p53 and Sp1, in the activation of cell-cycle arrest and apoptosis genes [16]. Pulldown studies determined the C-terminal and ODs of p53 as necessary for the interaction with Sp1 and activation of its target genes p21 and PUMA [16]. Other works attributed a repressive function to the C-terminal of the α-isoforms, giving an explanation to the higher activity of β over α-isoforms on p53-responsive elements [25]. In the present work we show that, although the SAM domain is dispensable for the transactivation of ∆Np73-α through Sp1 sites, both the extreme C-terminal and specially the OD are necessary for this transactivation. This explains why ∆Np73-β is less active in this model and supports a role for the OD in the interaction with Sp1. Moreover, truncated proteins harbouring the OD exert dominant negative actions over the full length ∆Np73-α protein, further supporting this idea. This observation agrees with those of Irwin et al. [26] made on consensus p53 responsive elements. Interestingly, in our model the distinctive 13 C-terminal residues of ∆Np73-α show some repressive function. There is promoter specificity for these function since the repressive behaviour on the NOXA promoter was exacerbated (data not shown). This observation differs from previous works attributing transactivation functions to those residues when acting through p53 responsive elements [7].

Finally, our work shows how the oncogene HRasV12 blocks the transactivation potential of ∆Np73-α. It has been reported that Ras and the oncogenic activity of ∆Np73 co-operate in the transformation of primary fibroblasts *in vitro* [27]. We were curious about the effect of Ras over the apoptosis promoting function of ∆Np73-α, so we cotransfected them along with the BIK promoter and, somehow expectedly, we observed that Ras impairs this function, acting as the oncogene it is. The concept that Ras needs to block ∆Np73-α transactivation to transform cells, further supports the new putative anti-tumoural function of this later. A Ras-activated kinase, ERK, phosphorylates Sp1 at domains involved in the interaction with p53 family members that may interfere the transactivation induced by ∆Np73 [16,28]. On the other hand, oncogenic Ras has been reported to differentially stabilize TAp73α [29], an isoform that can interact with ∆Np73α and may be blocking its capacity to transactivate BIK. Further experiments are needed to clarify the exact mechanism.

In summary, we report in the present study a new function of ∆Np73-α as apoptosis inducer through the co-ordinate activation of several proapoptotic genes. This activation is mediated by GC-rich elements in the promoter of these genes and requires the co-operation with Sp1, but the sequence diverges from that of a basic Sp1 responsive element. Thus, the final activation or repression of a certain gene is very dependent on the promoter context. This observation puts the focus on those tumours overexpressing both TA and ∆N isoforms of p73 and adds complexity to the possible outcomes when treating them.

## Abbreviations

∆Np73: N-terminus-truncated p73 isoform
B2M: β2 microglobulin
BH3: Bcl-2 homology domain 3
BIK: Bcl-2-interacting killer
FA: Fanconi anaemia
FANC: FA gene
MMC: mitomycin C
OD: oligomerization domain
PUMA: p53 up-regulated modulator of apoptosis
RLA: relative luciferase/galactosidase activity
RT: reverse transcription
SAM: sterile α motif
Sp1: specificity protein 1
TA: transactivation
TAp73: p73 containing the TA domain
